# Recto-vaginal fistulas: A case series

**DOI:** 10.1016/j.ijscr.2020.05.059

**Published:** 2020-05-30

**Authors:** Imad Ziouziou, Safaa Ammouri, Mohammed Ouazni, Harrison Sumba, Abdellatif Koutani, Ahmed Iben Attya Andaloussi

**Affiliations:** a Service d’urologie, CHU d’Agadir, Agadir, Morocco; b Equipe de recherche en médecine translationnelle et épidémiologie, Laboratoire des sciences de la santé, Faculté de médecine et de pharmacie, Université Ibn Zohr, Agadir, Morocco; c Service de gynécologie-obstétrique et d’endoscopie gynécologique, Maternité Soussi, CHU Ibn Sina, Faculté de médecine et de pharmacie, Université Mohamed V, Rabat, Morocco; d Service de chirurgie viscérale, CHU d’Agadir, Faculté de médecine et de pharmacie, Université Ibn Zohr, Agadir, Morocco; e Service d’urologie B, CHU Ibn Sina, Faculté de médecine et de pharmacie, Université Mohamed V, Rabat, Morocco

**Keywords:** Rectovaginal fistula, Martius, Falandry, Incontinence

## Abstract

**Introduction:**

Rectovaginal fistula (RVF) is defined as an abnormal communication between the anterior wall of the rectum and the posterior wall of the vagina. Many surgical techniques have been described in the treatment of RVF. However, none has proved its superiority. The aim of the study was to evaluate the functional results of surgical treatment of RVF using Martius and Falandry techniques in order to assess the feasibility and the efficacy of these techniques which were first described for vesico-vaginal fistulas.

**Methods:**

The study was a retrospective case series conducted in a single centre: Department of general surgery at Ibn Sina University Hospital in Rabat. We included patients with rectovaginal fistula consecutively recruited from 2011 to 2014. 10 patients developed RVF after surgery for rectal cancer (9 cases), uterine cancer (1 case). One patient had RVF for ano-rectal malformation. Colostomy was performed before the treatment of fistula in 9 cases (82 %). They underwent surgical treatment using Falandry (8 patients) and Martius techniques (3 patients) performed by an experienced urologist surgeon.

**Results:**

No postoperative complications were recorded. Time to discharge was postoperative day 3–4. There was a complete disappearance of RVF in 8 patients (72.7 % of cases), relapse in 2 cases (18 %), and failure in one case (9%). The average follow-up was 12.6 +/-10 months. Functionally, no long-term cases of fecal incontinence or dyspareunia were noted.

**Conclusion:**

The choice of surgical technique in the treatment of RVF remains difficult because of poor literature data and absence of consensus. RVF repair results either by Martius or Falandry techniques are encouraging with low morbidity.

## Highlights

• Falandry and Martius’ techniques were used firstly for vesico-vaginal fistulas with satisfying long-term functional results.
• The same techniques are feasible and safe for rectovaginal fistula repair.
• In this case series, patients had an improvement of their self-image and health-related quality of life.
• Temporary colostomy is recommended before this reconstructive surgery.

## 1 Introduction

Rectovaginal fistula (RVF) is defined as an abnormal communication between the anterior wall of the rectum and the posterior wall of the vagina. It’s said to be high when it’s above the anal sphincter [1]. RVFs are classed according to their location from the ano-rectal sphincter complex: ano-vaginal, recto-vaginal and reservoir/vaginal.

Many surgical techniques have been described in the treatment of RVF. However, none has proved its superiority over others. The choice of technique must consider location, size and characteristics of the fistula. Often the repair procedure is preceded by fecal stoma to reduce intra-rectal pressure and the risk of sepsis.

The aim of this study was to evaluate the functional results of surgical treatment of RVF using Martius and Falandry techniques in order to assess the feasibility and the efficacy of these techniques which were first described for vesico-vaginal fistulas.

We discuss in this article, through a retrospective study of 11 patients who had RVF in relation to an oncological context, therapeutic and evolutionary aspects of this pathology.

## 2 Methods

TShe study was a retrospective case series conducted in a single centre: Department of general surgery at Ibn Sina University Hospital in Rabat. We included patients with RVF consecutively recruited from 2011 to 2014. 9 patients developed RVF after surgery for rectal cancer: preoperative radiotherapy and anterior resection of the rectum.

One patient had total hysterectomy for uterine cancer. One patient had been operated on in childhood for anorectal malformation and having kept a residual RVF despite colostomy performed several years earlier (Table 1).

**Table 1:** medical history of patients.

	Age (ys)	Initial stage of cancer	Pre-operative radiotherapy	Surgery	Adjuvant chemotherapy	Oncologic evolution
*Patient 1*	45	localized	received	low anterior resection of rectal cancer + CAA*	received	no local, regional or distant recurrence
*Patient 2*	52	localized	received	low anterior resection of rectal cancer + CAA	not received	no local, regional or distant recurrence
*Patient 3*	47	localized	received	low anterior resection of rectal cancer + CAA	not received	métastase hépatique (metastasectomy + chemotherapy)
*Patient 4*	63	localized	received	low anterior resection of rectal cancer + CAA	not received	no local, regional or distant recurrence
*Patient 5*	43	localized	received	low anterior resection of rectal cancer + CAA	not received	no local, regional or distant recurrence
*Patient 6*	56	localized	received	low anterior resection of rectal cancer + CAA	not received	no local, regional or distant recurrence
*Patient 7*	49	localized	received	low anterior resection of rectal cancer + CAA	not received	no local, regional or distant recurrence
*Patient 8*	57	localized	not received	radical hysterectomy	not received	no local, regional or distant recurrence
*Patient 9*	46	localized	received	low anterior resection of rectal cancer + CAA	not received	no local, regional or distant recurrence
*Patient 10*	58	localized	not received	low anterior resection of rectal cancer + CAA	not received	no local, regional or distant recurrence
*Patient 11*	37	non oncologic context: anorectal malformation with rectovaginal fistula				

* ACA: colo-anal anastomosis.

All the patients reported gas leak and/or stool discharge per vagina.

Clinical examination diagnosed RVF and specified their location and sizes.

There was no anal sphincter incontinence in any of our patients.

They were treated for RVF using Falandry (8 patients) and Martius (3 patients) surgical techniques.

Colostomy was performed before the treatment of fistula in 9 cases (82 %).

Surgical treatment of RVF was performed by a urologist surgeon having more than 30 years experience particularly in vesicovaginal fistula repair by Falandry and Martius techniques. The mean follow-up duration was 12.6 months.

**Martius technique**

Under spinal or general anesthesia, the patient is placed in modified lithotomy position.

Vaginal valves allow endo-vaginal exposure. Saline or better Xylocaine with epinephrine may be injected in the rectovaginal septum to facilitate dissection and hemostasis. Vaginal excision starts with excision of the vaginal fistula orifice and continues obliquely down and to the left without injury to the anal sphincter. Antero-posterior incision is made on the relief of the left labia majora.

Then, the rectovaginal septum is dissected from both sides over a width of about 4 cm to facilitate the passage of the labial adipose flap.

After abrasion of the intra-rectal fistula orifice, the rectal wall is repaired transversely using absorbable 4-0 PDS sutures.

From the labial incision, subcutaneous dissection is performed to create a sufficiently wide tunnel between the two incisions.

The labial adipose flap is passed through the created tunnel to cover the front rectal mucosa.

Several separate absorbable 4-0 PDS sutures allow to keep in place the adipose flap. Careful hemostasis is done. Finally, the vaginal mucosa is closed using 3-0 PDS and the wound using non absorbable 3-0 separate suture with a Redon drain placed in the tunnel and adipose harvest site.

**Falandry technique**

**Fig. 1:**
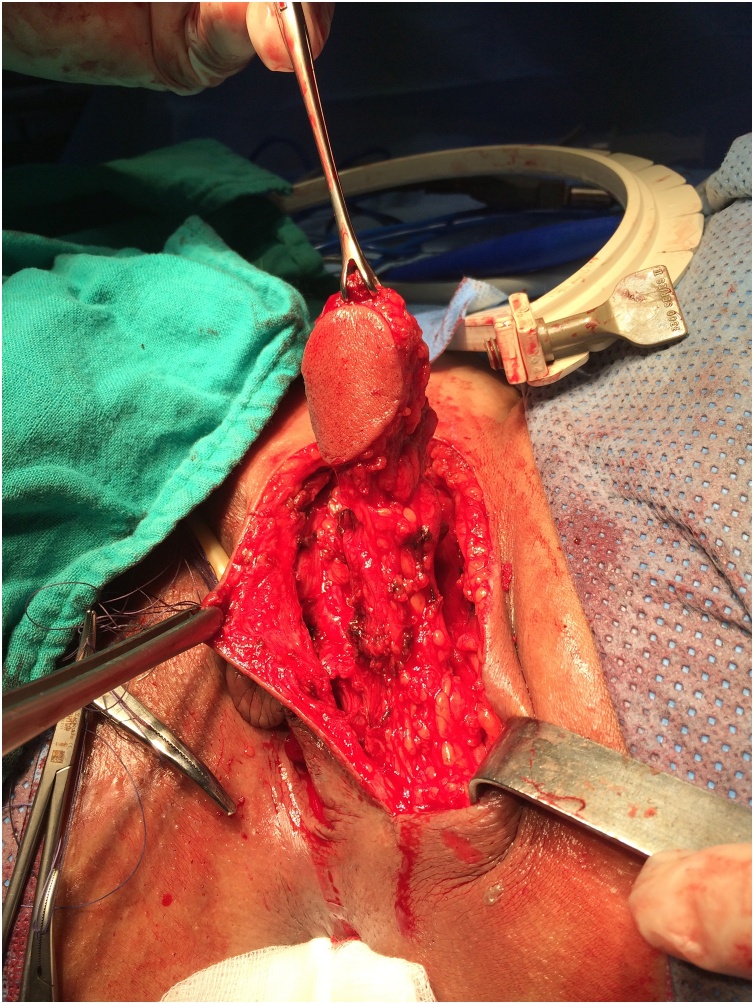
dissection of pedicled labial skin flap of 4 to 5 cm long and 1.5 to 2 cm.

**Fig. 2:**
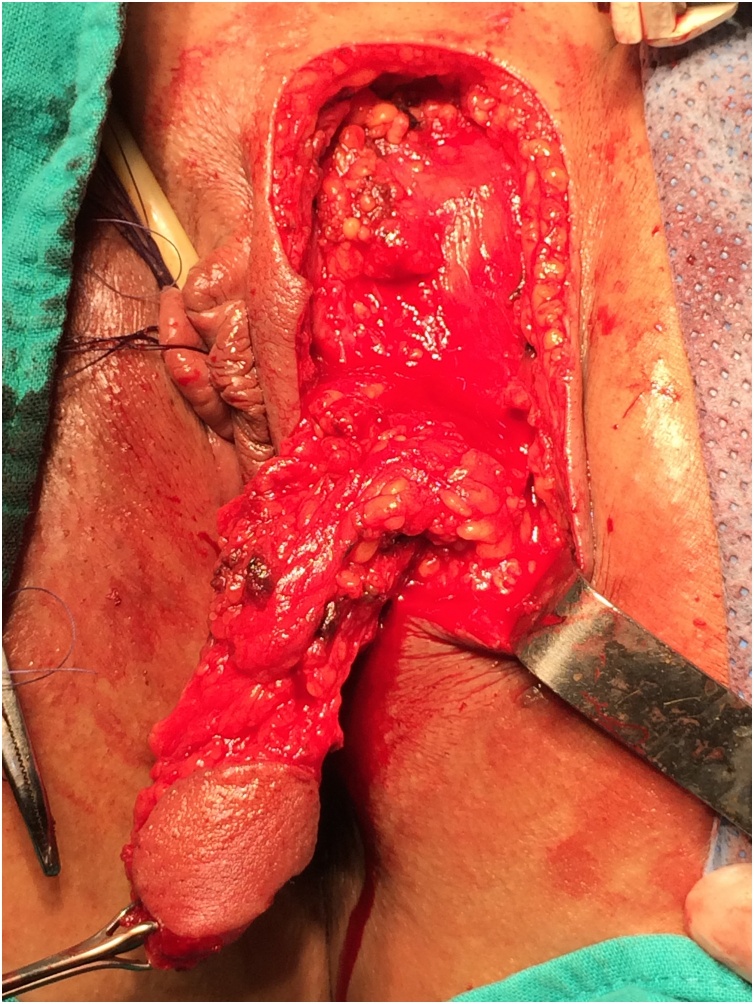
after completion of dissection, sufficiently long flap is obtained allowing mobilization without tension.

**Fig. 3:**
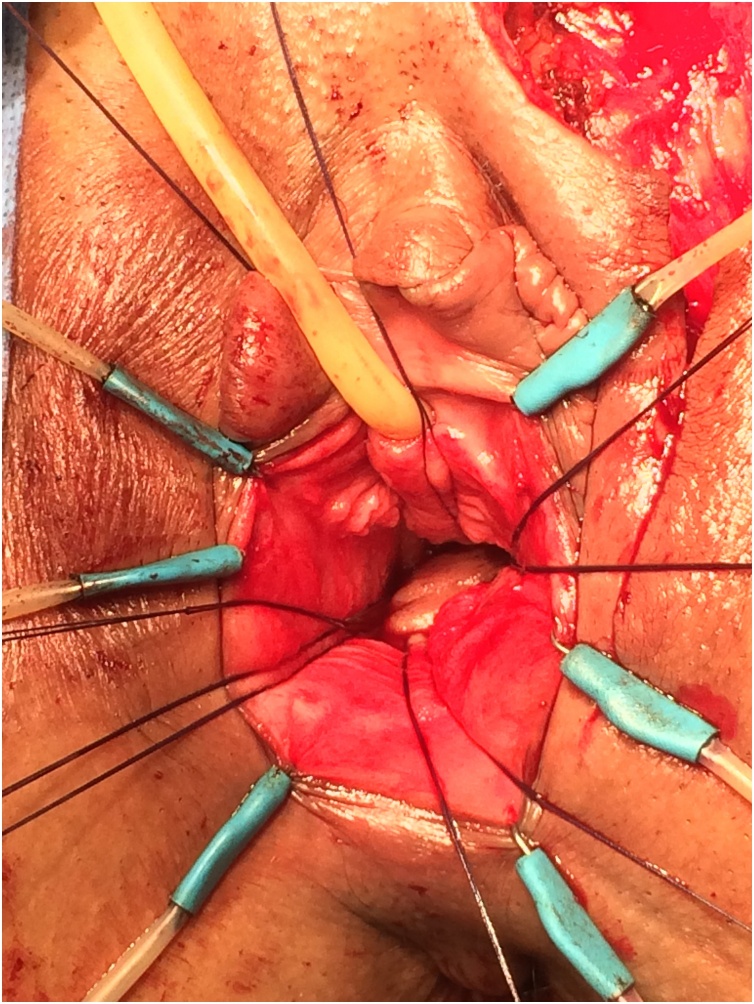
the flap is attached to the rectal mucosa with absorbable sutures (PDS 4-0).

This intervention is conducted under spinal or general anesthesia, and involves taking a skin flap from the inner side of the left labium majus which will be inserted in rectovaginal. The incision isolates a labial skin flap of 4–5 cm long and 1.5–2 cm wide with a sufficiently long meso so as to reach the vaginal cavity without tension (Fig. 1, Fig. 2). The time of exposure of the fistula and the creation of subcutaneous tunnel between the labial and vaginal incisions are similar to those described above in the previous technique. The skin graft is mobilized beside the fistula by the subcutaneous tunnel, and then attached to the rectal mucosa with absorbable separate 4-0 PDS suture (Fig. 3). Vaginal mucosa and labial skin closured is done after careful hemostasis.

The research work has been reported in line with the PROCESS criteria [2].

## 3 Results

The mean age of patients was 50.27 +/− 7.68 years.

The mean duration of the procedure was 1 h. Bleeding was minimal in all the cases. No blood transfusion was required in any of the cases.

Postoperative period was uneventful in all cases. No immediate postoperative complications were recorded.

The mean time to first flatus was 24 h. First ambulation started at postoperative day1. Time to discharge was day 3–4.

The complete disappearance of RVF was immediately obtained in 8 patients, 72.7 % of cases. The average follow-up was 12.63 +/- 10.03 months.

Functionally, no case of anal incontinence or dyspareunia was noted in the long run.

Repair using Falandry technique failed in a case of a patient whose oncological status was pejorative hindering a second surgery attempt.

A residual fistula was seen in a patient operated on using Falandry technique without colostomy. This complication was managed by performing colostomy with as result: spontaneous closure of the fistula.

Recurrence of RVF was observed in a patient operated on using Falandry technique, a month after surgery. She secondarily had suture of the fistula with remarkable evolution thereafter (Table 2).

**Table 2:** Fistula’s characteristics, treatment and outcome.

	Fistula diameter	Localization of the fistula - Distance to the vulva (cm)	Stoma	Corrective surgical technique	Long-term outcome	Follow-up (months)
*Patient 1*	2 cm	5	colostomy before corrective surgery	Falandry	successful	24
*Patient 2*	1 cm	6	colostomy before corrective surgery	Falandry	successful	18
*Patient 3*	2 cm	5	colostomy before corrective surgery	Falandry	failed, not re-operated because of the pejorative oncologic evolution	2
*Patient 4*	2 cm	5	colostomy after residual fistula	Falandry	spontaneous closure of the residual fistula after colostomy	36
*Patient 5*	1 cm	3	not performed	Martius	successful	12
*Patient 6*	2 cm	6	colostomy before corrective surgery	Falandry	successful	10
*Patient 7*	3 cm	4	colostomy before corrective surgery	Falandry	successful	11
*Patient 8*	2 cm	3	colostomy before corrective surgery	Martius	successful	5
*Patient 9*	2 cm	2	ileostomy before corrective surgery	Martius	successful	3
*Patient 10*	3 cm	1	ileostomy before corrective surgery	Falandry	recurrence at 1 month re-operated with good outcome	10
*Patient 11*	6 mm	3	colostomy 3 years before corrective surgery	Falandry	successful	8

## 4 Discussion

The circumstances of occurrence of RVF may be post-operative, post-obstetric and traumatic. The most frequent cause of RVF is obstetrical trauma related to prolonged labor with ischemic injury that leads to tissue necrosis and formation of fistula [3,4].

The second most common cause is Crohn disease [5].

Other causes include pelvic irradiation, malignancies, and postsurgical complications [6]. RVF may be secondary to anterior resection of the rectum with colorectal anastomosis (incidence: 0.1–2.9 %) [7,8]. Causes include technical errors during anastomosis, misapplication of stapling device injuring the posterior wall of the vagina, as well as anastomotic ischemia. Moreover, there are cases reported in the literature of spontaneous RVF secondary to bevacizumab [[9], [10], [11]].

Clinically, RVF manifests by passage of liquid stool or gas per vagina. In addition, some patients may have dyspareunia, vaginal irritation, peri-anal pain and recurrent vaginitis and cystitis [7].

Among these factors, two were present in all of our patients: cancer (10 cases of rectal and 1 case of uterine cancer) and pelvic surgery.

Rothenberger et al. classified RVFs as simple and complex fistulas [12]:

- Simple RVFs are located in the lower third and middle of the vagina; with a diameter less than 2.5 cm and are typically caused by trauma or infection.
- Complicated RVFs are located in the upper third of the vagina; their diameter is greater than 2.5 cm and are caused by chronic inflammatory bowel disease, radiation or tumor.

Most patients with RVF complain of flatulence, passage of gas leak and/or stool per vagina, recurrent urinary tract infections, vaginal discharge or fetid leucorrhea [13].

Moreover, all of our patients had typical symptoms of RVF consisting of passage of gas and/or stool per vagina.

Diagnosis is done by questioning and physical examination. Clinical examination was sufficient to retain RVF diagnosis in our patients and characterize the fistula: size, location, surrounding tissue. In case of diagnostic doubt or as part of an etiological investigation, there may be use of colonoscopy, endo-anal magnetic resonance imaging, computed tomography, rectal ultrasound, and opacification: proctography, vaginography, defecography [[14], [15], [16]].

RVF repair by abdominal procedure has been proposed by some authors, in cases of high fistula above the dentate line. Treatment of RVF in this case consists of resection of the rectum with colo-anal anastomosis or repair by transposition of tissues [[17], [18], [19], [20], [21], [22], [23]].

Perineal and trans-anal approach is preferred in cases of low RVF below the sphincter complex. Repair options include a flap repair of the anterior rectum and a mucosal flap with or without anal sphincter plasty [[24], [25], [26], [27], [28], [29], [30], [31], [32], [33], [34]].

In fact, there are various procedures used in RVF surgery with varying success rates, including pelvic floor proctectomy, fibrin glue, and interventions of tissue interposition [35,36].

Among these interventions of tissue interposition, Martius technique was used. Success rate of this technique reported in literature was between 65 and 100 % (Table 3).

**Table 3:** Series on Martius’ technique in rectovaginal fistulas.

Author	Year	Number of patients	Etiology	Rate of success
Kniery K [37]	2015	1	–	–
Reichert M [38]	2014	1	obstetrical	–
Kin C [39]	2012	5	obstetrical, post-hysterectomy, Crohn's disease	100 %
Pitel S [40]	2011	20	Crohn's disease, ulcerative colitis, post-surgery of bartholinitis, post-hemorroidectomy, post-surgery of rectocele, obstetrical, cryptoglandular, Hirschprung	65 %
McNevin MS [41]	2007	16	obstetrical, cryptocglandular, Crohn's disease	94 %
Songne K [42]	2007	14	Crohn's disease, ulcerative colitis, pelvic radiotherapy, obstetrical, villous tumor	100 %
White AJ [43]	1982	14	pelvic radiotherapy	78.5 %

Three patients in our series were treated using Martius technique including two after stoma and one without stoma, in whom result was healing in all three cases (100 %).

To our knowledge, we describe for the first time Falandry technique in RVF repair. This surgical technique was described in the early 1990s by Falandry, who used pedicled skin of the labia majora as tissue interposition in contexts of complicated post-obstetrics urethro-vaginal and vesico-vaginal fistulas repair [[44], [45], [46]].

We used the technique in 8 patients and the result was successful in 5 patients (62.5 %), recurrence a month later in one patient (12.5 %) which was treated by performing a colostomy, residual persistence of fistula in one patient (12.5 %) which was treated with colostomy leading to spontaneous closure. Treatment with Falandry technique failed in only one patient (12.5 %) who was not re-operated due to an adverse oncologic evolution.

## 5 Conclusion

The choice of surgical technique in the treatment of RVF remains difficult because of poor literature data and absence of consensus. Results on RVF repair either by Martius or Falandry technique are encouraging with low morbidity.

## Declaration of Competing Interest

All the authors declare having no conflict of interest.

## Sources of funding

This research did not receive any grant.

## Ethical approval

As per local institutional report guidelines: a case series does not qualify as a clinical investigation. It was not mandated to be subject to approval by ethics committee.

## Consent

Written informed consent was obtained from the patients for publication of this study with accompanying images.

Copies of the written consents are available for review by the Editor-in-Chief of this journal.

## Author contribution

Imad Ziouziou, Safaa Ammouri: Conceptualization, Methodology, Writing.

Mohammed Ouazni: Investigation, reviewing Harrison Sumba: Reviewing.

Ahmed Ibn Attya Andaloussi: Supervision.

Abdellatif Koutani: Conceptualization, Reviewing, Supervision.

## Registration of research study

This study was registered at:

1 Researchregistry: https://www.researchregistry.com.
2 Registration ID: researchregistry5514.
3 https://www.researchregistry.com/browse-the-registry#home/registrationdetails/5e983c0ae621b200159c8f05/

## Guarantor

Imad Ziouziou.

## Provenance and peer review

Not commissioned, externally peer-reviewed.
